# Subclinical pulmonary congestion in ST-segment elevation myocardial infarction assessed by computed tomography

**DOI:** 10.3389/fradi.2026.1800804

**Published:** 2026-04-01

**Authors:** Yufan Gao, Keyi Cui, Shuo Liang, Minghui Hua, Hong Zhang, Zhigang Guo

**Affiliations:** 1 Academy of Medical Engineering and Translational Medicine, Tianjin University, Tianjin, China; 2 Department of Radiology, Chest Hospital, Tianjin University, Tianjin, China; 3 Department of Cardiac Surgery, Chest Hospital, Tianjin University, Tianjin, China; 4 Tianjin Key Laboratory of Cardiovascular Emergency and Critical Care, Tianjin Municipal Science and Technology Bureau, Tianjin, China

**Keywords:** extravascular lung water, myocardial infarction, prognosis, risk stratification, tomography, x-ray computed

## Abstract

**Objectives:**

To evaluate the prognostic value of subclinical pulmonary congestion, assessed as extravascular lung water (EVLW) by computed tomography (CT), in ST-segment elevation myocardial infarction (STEMI) patients with Killip class 1.

**Methods:**

This retrospective study included Killip class 1 STEMI patients who underwent CT prior to primary percutaneous coronary intervention (PCI). EVLW was derived from mean lung density. The Global Registry of Acute Coronary Events (GRACE) score was calculated. The endpoint was in-hospital major adverse cardiovascular events (MACE), defined as all-cause mortality, acute heart failure, cardiogenic shock, resuscitated cardiac arrest, or stroke. The predictive improvement of adding EVLW to the GRACE score was assessed using receiver operating characteristic (ROC) analysis, net reclassification improvement (NRI), and integrated discrimination improvement (IDI).

**Results:**

Among 249 patients (mean age 59 ± 11 years; 195 men), 28 experienced MACE. Patients with MACE had a higher GRACE score (144.96 ± 22.95 vs. 133.63 ± 19.84, *P* = 0.006) and EVLW (24.24% vs. 21.36%, *P* = 0.001). Adding EVLW to the GRACE score significantly increased the area under the ROC curve (AUC) (0.754 vs. 0.656, *P* = 0.045), NRI (0.491, *P* = 0.013), and IDI (0.060, *P* = 0.019).

**Conclusions:**

In Killip class 1 STEMI patients, CT-identified subclinical pulmonary congestion is associated with an increased risk of in-hospital MACE.

## Introduction

Ischemic heart disease, including ST-segment elevation myocardial infarction (STEMI), is a common cause of death and disability worldwide (1). Heart failure (HF) is a frequent and severe complication of acute myocardial infarction (AMI) and is associated with worse in-hospital and short-term outcomes (2, 3). The prognosis of patients after myocardial infarction is highly heterogeneous (4) and several classifications have been reported to stratify the risk of patients with STEMI (5–7). Among these, the Killip classification, which is based on the severity of HF signs at admission, remains a simple and valuable risk stratification tool (8) and is an important part of the Global Registry of Acute Coronary Events (GRACE) risk score (7). Patients with Killip class 1 at admission have been reported to have a better prognosis than those with higher Killip classes (9, 10), largely due to primary percutaneous coronary intervention (PCI) being performed smoothly (11). However, some patients with Killip class 1 (without HF signs at admission) suffer from serious in-hospital complications, even after undergoing primary PCI (12). Identifying high-risk STEMI patients with Killip class 1 to make accurate treatment and diagnostic decisions remains a challenge.

Previous studies have demonstrated a subclinical increase in pulmonary fluid content in AMI without HF (13). Early signs of HF or pulmonary edema on physical examination or chest x-ray are not obvious or sensitive enough for effective detection of mild heart failure or subclinical pulmonary edema in STEMI patients with Killip class 1. Computed tomography (CT) allows for fast chest image acquisition with high spatial resolution and provides important information about subtle lung density changes. Recent studies have shown that CT provides a fully quantitative tool to calculate extravascular lung water (EVLW) based on mean lung density for subclinical congestion detection (14, 15). Jain et al. found higher ratios of EVLW to total lung volume in patients with HF with preserved ejection fraction compared with control subjects, despite the absence of clinically overt pulmonary congestion, suggesting a subclinical increase in EVLW (14). These findings suggest its value as a risk stratification tool in STEMI patients with Killip class 1 (without HF signs at admission).

The present study aimed to evaluate the incremental prognostic value of EVLW on top of the GRACE risk score in STEMI patients with Killip class 1 who underwent primary PCI.

## Methods

### Study population

This study was approved by the Ethics Committee of our hospital. Written informed consent was waived due to the retrospective nature of the study.

**Figure 1:**
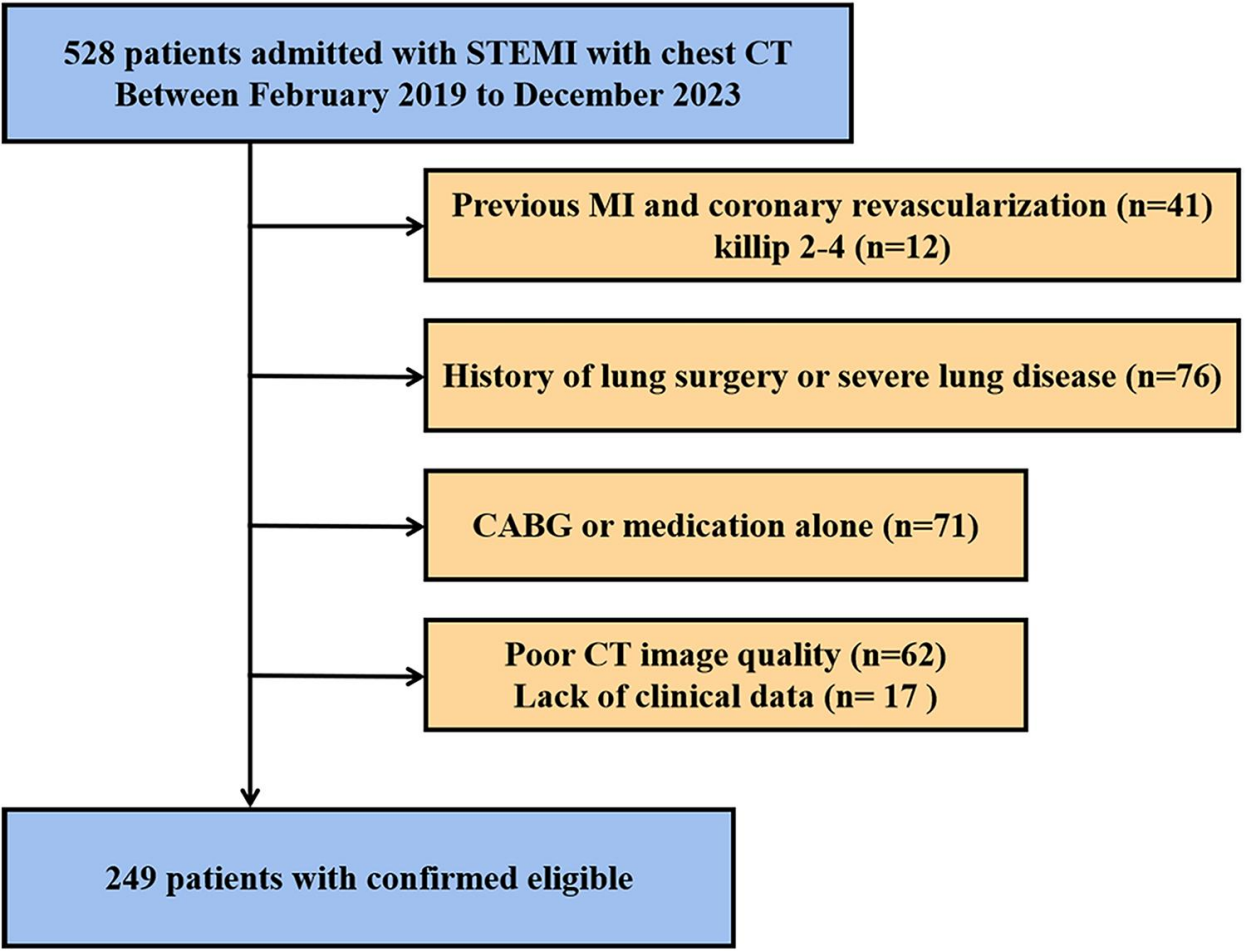
Flow chart of the present study. STEMI, ST-segment elevation myocardial infarction; CT, computed tomography; MI, myocardial infarction; CABG, coronary artery bypass graft.

The study retrospectively investigated 249 patients admitted with STEMI who underwent chest CT between February 2019 and December 2023 at Chest Hospital, Tianjin University. At our institution, a tertiary cardiothoracic center, chest CT is frequently performed for clinical indications such as evaluation of suspected aortic dissection, assessment of pulmonary comorbidities, or pre-procedural planning. Only patients classified as Killip class 1 (no signs of HF) who underwent non-contrast CT at admission were included. The median time interval from symptom onset to CT acquisition was 5 h (interquartile range: 3.5–6.5 h), and the median time from CT acquisition to primary PCI was 1 h (interquartile range: 0.9–1.4 h). The exclusion criteria were as follows: previous myocardial infarction; previous coronary revascularization; history of lung surgery; severe lung disease, such as severe pulmonary emphysema, pulmonary interstitial fibrosis or lung tumors; severe liver or kidney dysfunction; and poor image quality, such as motion or respiratory artifacts. Patients who underwent coronary artery bypass graft, or medical therapy only without revascularization were also excluded. A detailed flow chart of the present study is shown in Figure 1.

### CT acquisition

CT images were acquired on a 256-slice CT scanner (Phillips iCT, Philips Healthcare, Best, The Netherlands) at admission. CT images were obtained with the following parameters: tube voltage,120 kV; automatic tube current modulation; pitch, 0.993; matrix size, 512 × 512; rotation time, 0.5; collimation, 1.5 mm. Images were acquired during inspiratory breath hold in the supine position. All imaging was performed with a filtered back-projection reconstruction with a slice thickness of 1.5 mm and a reconstruction interval of 1.5 mm.

### Determination of EVLW and GRACE risk score

All CT images were imported into a commercial Chronic Obstructive Pulmonary Disease software (IntelliSpace Portal, Version 9.0, Philips Healthcare, Best, The Netherlands) which automatically segments the lungs and measures the mean lung density, with manual correction performed as necessary by two independent observers (Y.F.G. with 6 years of experience and K.Y.C. with 8 years of experience) who were blinded to clinical outcomes. EVLW quantification was performed using a validated quantitative method based on the exclusion of voxels not falling within the attenuation range of air (−1,000 HU) and water (0 HU), as previously described (15). Briefly, the mean lung density was measured within a lung region of interest after automated exclusion of identifiable blood vessels and airways using density thresholding. EVLW (%) was calculated using the following formula: EVLW (%) = [mean lung density (HU) + 1,000]/10. This quantitative approach allows for objective measurement of lung water content independent of subjective visual assessment. To assess intra- and inter-observer reproducibility, 20 randomly selected CT scans were reanalyzed by the same operator after a two-week interval and independently by a second operator.

The GRACE risk-prediction tool was previously described (7). The score is derived from several variables (age, heart rate, systolic blood pressure, serum creatinine level, Killip class, cardiac arrest at admission, ST-segment deviation, and elevated cardiac biomarkers) and calculated for each patient.

### Endpoint definition

The primary endpoint of the study was in-hospital major adverse cardiovascular events (MACE), which included all-cause mortality, acute HF, cardiogenic shock, resuscitated cardiac arrest, and stroke.

### Statistical analysis

Continuous variables were tested for normality using the Shapiro–Wilk test. Normally distributed continuous variables were expressed as mean ± standard deviation, while non-normally distributed continuous variables were reported as median and interquartile range. Independent-sample *t*-test was applied to assess normally distributed continuous variables, and Mann–Whitney *U* test was applied for non-normally distributed continuous variables. Chi-square tests were applied to assess categorical variables. Spearman rank correlation was performed to assess statistical associations between variables. Univariate and multivariate logistic regressions were performed to identify independent predictors for in-hospital MACE. Two logistic regression models were constructed (i.e., one with GRACE risk score alone and the other with the combination of EVLW). Discrimination was assessed with the area under the receiver operating characteristic (ROC) curve (AUC). DeLong's test was used to compare the AUC from each of the models (16). Net reclassification improvement (NRI) and integrated discrimination improvement (IDI) were performed to analyze the degree to which the addition of EVLW to the GRACE risk score model improved predictive ability (17). A *P*-value < 0.05 was considered statistically significant.

Statistical analyses were performed with R version 4.4.0 (R Foundation for Statistical Computing, Vienna, Austria).

## Results

### Patient characteristics

The baseline characteristics of the study population are summarized in Table 1. A total of 249 patients (59 ± 11 years) were included in the study. Among the 249 patients, 28 (11.2%) experienced in-hospital MACE. There were no significant differences in the prior history of hypertension and diabetes. Compared with the patients without MACE, those who experienced MACE had higher heart rate, white blood cell (WBC) count, and high-sensitivity C-reactive protein (hs-CRP) (all *P* < 0.05). Patients with MACE had lower left ventricular ejection fraction than those without MACE (*P* < 0.001). In addition, patients with MACE had higher N-terminal pro-B-type natriuretic peptide (NT-proBNP). Compared with the patients without MACE, those who experienced MACE had higher GRACE risk score (*P* = 0.006).

**Table 1:** Baseline characteristics.

Variable	All patients (*n* = 249)	With MACE (*n* = 28)	Without MACE (*n* = 221)	*P* value
Age (years)	59.04 ± 10.69	61.82 ± 11.43	58.69 ± 10.57	0.145
Male, *n* (%)	195 (78.3)	20 (71.4)	175 (79.2)	0.348
Hypertension, *n* (%)	142 (57.0)	16 (57.1)	126 (57.0)	0.990
Smoker, *n* (%)	171 (68.7)	18 (64.3)	153 (69.2)	0.595
Diabetes, *n* (%)	71 (28.5)	7 (25.0)	64 (29.0)	0.662
SBP (mmHg)	134.29 ± 17.69	130.18 ± 20.58	134.81 ± 17.28	0.193
Heart rate (beats/min)	71.92 ± 12.12	77.25 ± 13.86	71.25 ± 11.75	0.013
LVEF (%)	51.00 (46.00, 56.00)	45.00 (36.50, 50.00)	52.00 (47.00, 56.00)	<0.001
Laboratory index				
Hs-TnT (ng/mL)	4.31 (1.99, 9.34)	10 (4.52, 10)	4.02 (1.84, 8.59)	<0.001
CK (U/L)	1,351.00 (609.50, 2,756.50)	2,560.00 (1,313.75, 4,238.00)	1,292.00 (569.50, 2,601.00)	<0.001
CK-MB (U/L)	148.00 (66.00, 269.00)	202.50 (138.75, 517.25)	143.00 (62.00, 238.50)	0.002
LDH (U/L)	549.00 (339.00, 863.50)	923.50 (667.75, 1,207.25)	518.00 (331.50, 815.50)	<0.001
ALT (U/L)	41.70 (27.98, 62.30)	67.35 (40.38, 96.38)	39.05 (27.20, 57.25)	<0.001
AST (U/L)	156.60 (79.25, 274.30)	278.35 (160.35, 385.55)	142.60 (73.80, 257.45)	0.001
NT-proBNP (pg/mL)	685.90 (287.70, 1,453.00)	1,637.50 (701.20, 2,969.50)	650.35 (257.33,1,368.75)	<0.001
WBC (×10^9^ /L)	9.83 (8.06, 11.74)	11.74 (8.89, 14.12)	9.72 (8.02, 11.51)	0.024
Hematocrit (%)	42.00 (38.30, 44.65)	39.70 (37.05, 45.18)	42.10 (38.55,44.60)	0.248
Creatinine (umol/L)	73.00 (62.50, 84.50)	82.50 (69.00, 93.50)	72.00 (62.00, 82.00)	0.012
Hs-CRP (mg/L)	5.71 (2.86, 12.70)	12.04 (5.26, 43.54)	5.46 (2.57, 10.99)	0.001
eGFR (mL/min/1.73 m^2^)	94.94 (82.07, 102.39)	85.88 (55.25, 94.87)	95.63 (85.49, 103.11)	0.004
Multivessel disease	176 (70.7)	21 (75.0)	155 (70.1)	0.594
Infarct-related artery				
Left main trunk	2 (0.8)	0 (0.0)	2 (0.9)	1.000
LAD	129 (51.8)	19 (67.9)	110 (49.8)	0.071
LCX	38 (15.3)	0 (0.0)	38 (17.2)	0.035
RCA	95 (38.2)	10 (35.7)	85 (38.5)	0.778
Culprit lesion location				
Proximal lesion	126 (50.6)	16 (57.1)	110 (49.8)	0.462
GRACE risk score	134.90 ± 20.48	144.96 ± 22.95	133.63 ± 19.84	0.006
EVLW (%)	21.62 (18.55, 25.51)	24.24 (21.36, 28.79)	21.36 (18.30, 25.27)	0.001

Data are mean ± SD, *n* (%), or median (interquartile range). MACE, major adverse cardiovascular events; SBP, systolic blood pressure; LVEF, left ventricle ejection fraction; hs-TnT, high-sensitivity troponin T; CK, Creatine Kinase; CK-MB, Creatine Kinase-Muscle and Brain; LDH, lactate dehydrogenase; ALT, alanine transaminase; AST, aspartate transaminase; WBC, white blood cell; hs-CRP, high-sensitivity C-reactivity protein; NT-proBNP, N-terminal pro-B-type natriuretic peptide; eGFR, estimated glomerular filtration rate using the CKD-EPI formula; LAD, left anterior descending coronary artery; LCX, left circumflex coronary artery; RCA, right coronary artery; GRACE, Global Registry of Acute Coronary Events; EVLW, extravascular lung water. Normal reference ranges: WBC, 3.5–9.5 × 10^9^ /L; hs-TnT, <0.014 ng/mL; NT-proBNP, <300 pg/mL; CK, 40–200 U/L; CK-MB, <25 U/L; LDH, 120–250 U/L; ALT, 7–40 U/L; AST, 13–35 U/L; Hematocrit, 35%–45%; Creatinine, 41–81 μmol/L; hs-CRP, ≤5 mg/L. NT-proBNP was unavailable for 9 patients (*n* = 240 for this analysis).

### EVLW determined by CT

**Figure 2:**
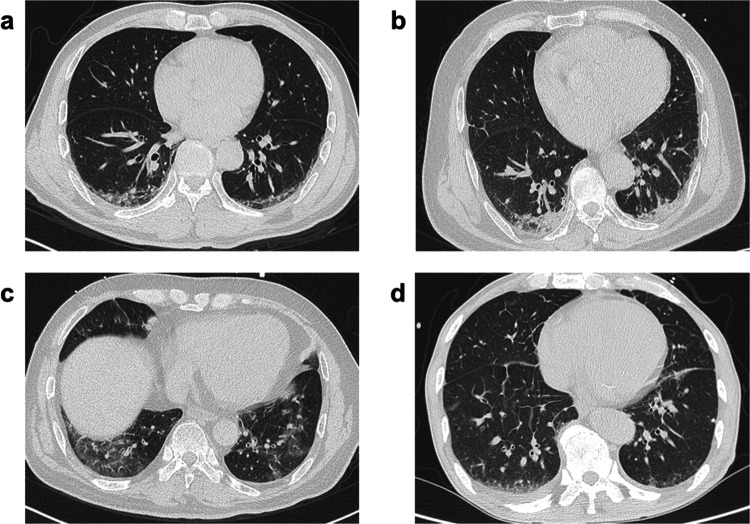
Representative chest CT images from the study cohort. **(a)** Subpleural ground-glass opacity. **(b)** Subpleural consolidation. **(c)** Diffuse ground-glass opacity with interlobular septal thickening. **(d)** Interlobular septal thickening.

Compared to patients without MACE, patients who experienced MACE had an increased EVLW [24.24% [21.36, 28.79%] vs. 21.36% [18.30, 25.27%]; *P* = 0.001; Table 1]. Among patients with elevated EVLW, various CT imaging findings were observed, including subpleural ground-glass opacity, subpleural consolidation, pulmonary ground-glass opacity, and interlobular septal thickening. Figure 2 shows representative cases illustrating these findings. Table 2 shows the correlations between EVLW and other variables. For all STEMI patients in this cohort, EVLW demonstrated significant positive correlations with a range of biomarkers indicative of myocardial damage and inflammation, including high-sensitivity troponin T (hs-TnT), creatinine kinase, Creatine Kinase-Muscle and Brain (CK-MB), lactate dehydrogenase, alanine transaminase, aspartate transaminase, and WBC count, with all associations being highly statistically significant (*P* < 0.001). Furthermore, EVLW showed a notable positive association with NT-proBNP level. Conversely, EVLW exhibited a significant negative correlation with hematocrit.

**Table 2:** Correlations between EVLW and biomarkers.

Variable	*r*	*P* value
Hs-TnT (ng/mL)	0.254	<0.001
CK (U/L)	0.312	<0.001
CK-MB (U/L)	0.317	<0.001
LDH (U/L)	0.282	<0.001
ALT (U/L)	0.291	<0.001
AST (U/L)	0.302	<0.001
NT-proBNP (pg/mL)	0.146	0.024
WBC (×10^9^ /L)	0.273	<0.001
Neutrophil (×10^9^ /L)	0.254	<0.001
Lymphocyte (×10^9^ /L)	0.072	0.260
Monocyte (×10^9^ /L)	0.015	0.818
Hematocrit (%)	−0.142	0.025

EVLW, extravascular lung water; hs-TnT, high-sensitivity troponin T; CK, Creatine Kinase; CK-MB, Creatine Kinase-Muscle and Brain; LDH, lactate dehydrogenase; ALT, alanine transaminase; AST, aspartate transaminase; WBC, white blood cell; NT-proBNP, N-terminal pro-B-type natriuretic peptide. NT-proBNP was unavailable for 9 patients (*n* = 240 for this analysis).

### Improvement in risk prediction during hospitalization

**Figure 3:**
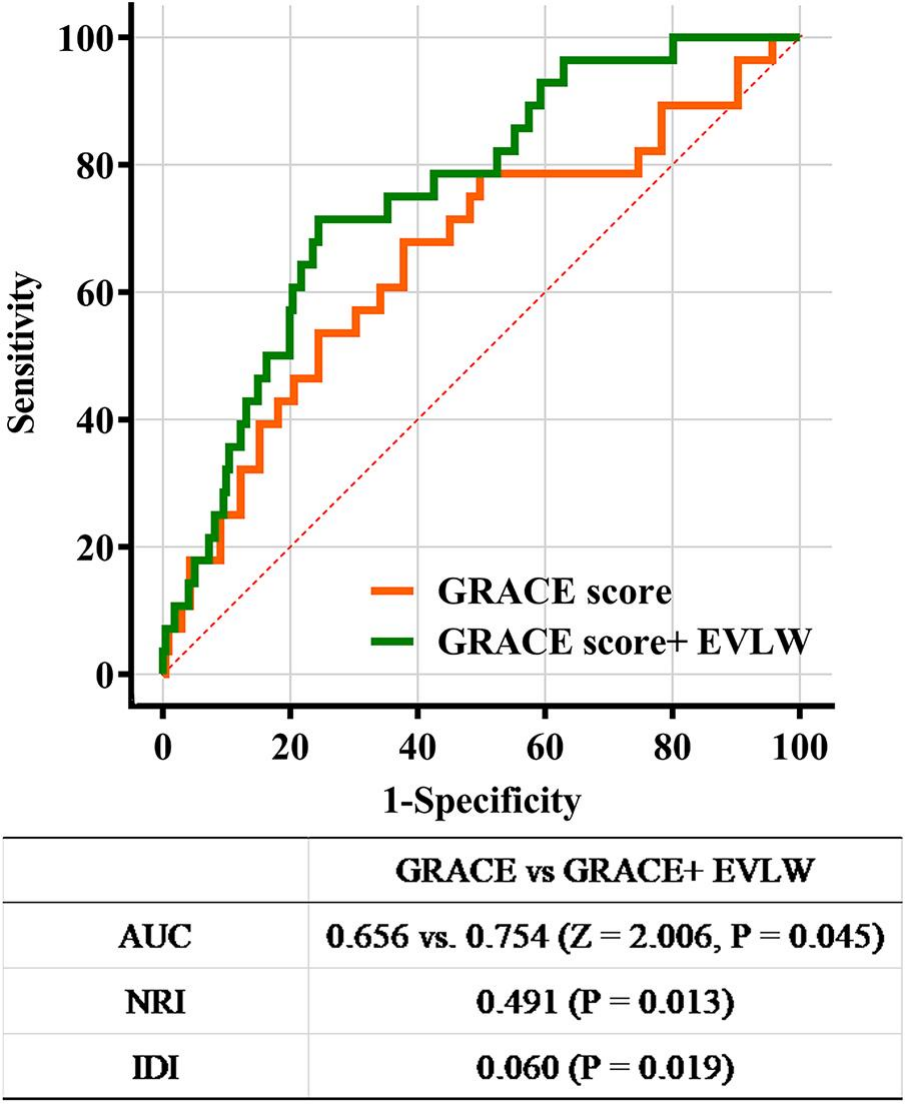
Receiver operating characteristic curve analysis. GRACE, global registry of acute coronary events; EVLW, extravascular lung water; AUC, area under the receiver operating characteristic curve; NRI, net reclassification improvement; IDI, integrated discrimination improvement.

The GRACE score was 134.90 ± 20.48 in overall population. Table 3 summarizes the results of the univariate and multivariate logistic regression analysis for prediction of in-hospital MACE. The GRACE risk score and EVLW were associated with increased MACE risk (all *P* < 0.05) in univariable logistic regression analysis. The EVLW was still an independent predictor of MACE after adjusting for GRACE score (odds ratio = 1.164, 95% confidence interval = 1.067–1.270; *P* = 0.001). In the area under ROC analyses, the AUC significantly increased after the addition of EVLW to the GRACE score (0.754 vs. 0.656, Z = 2.006; *P* = 0.045; Figure 3). Moreover, the inclusion of EVLW into the GRACE score model was associated with an NRI of 0.491 (*P* = 0.013), suggesting an effective reclassification. The IDI again showed that the diagnostic performance of the model was significantly improved (IDI = 0.060; *P* = 0.019; Figure 3).

**Table 3:** Univariate and multivariable logistic regression analysis for in-hospital MACE predictors.

	Univariate		Multivariate	
Variable	OR (95% CI)	*P* value	OR (95% CI)	*P* value
GRACE risk score	1.028 (1.008–1.049)	0.007	1.028 (1.007–1.050)	0.010
EVLW (%)	1.164 (1.071–1.265)	<0.001	1.164 (1.067–1.270)	0.001

MACE, major adverse cardiovascular events; CI, confidence interval; OR, odds ratio; GRACE, global registry of acute coronary events; EVLW, extravascular lung water.

### Reproducibility of EVLW measurements

Intra-observer reproducibility was excellent, with an ICC of 0.999 (95% CI: 0.997–0.999). Inter-observer reproducibility was also excellent, with an ICC of 0.979 (95% CI: 0.948–0.991).

## Discussion

In this study, we detected and assessed subclinical pulmonary congestion based on increased EVLW using chest CT in STEMI patients with Killip class 1. Our findings revealed significant positive correlations between EVLW and indices of myocardial damage, including hs-TnT, CK-MB, and NT-proBNP. EVLW also exhibited significant correlations with WBC count and hematocrit. Patients with MACE had an increased EVLW compared to those without MACE. Moreover, EVLW was an independent predictor of in-hospital MACE and provided incremental value to the GRACE score in predicting in-hospital MACE.

The Killip classification has been used to stratify the risk of patients with AMI for more than 40 years (8) and is included in the GRACE risk score. Although the majority of STEMI patients are categorized as Killip class 1 upon admission—a group traditionally associated with favorable in-hospital prognosis (2)—a subset of these patients still exhibit considerable risk of adverse outcomes, highlighting the need for enhanced risk stratification (2). One study examined the evolving changes in lung interstitial fluid content after first AMI and proposed that altered fluid translocation from the intravascular to alveolar interstitial compartment was not unusual in AMI without congestive HF (13). Shochat et al. demonstrated that lung impedance-guided pre-emptive therapy in STEMI patients with Killip class 1 decreased the incidence of in-hospital pulmonary edema and improved short- and long-term outcomes (18). More recently, subclinical congestion detected by lung ultrasound was associated with in-hospital prognosis of STEMI (19). Our study extends these findings by using chest CT to directly quantify EVLW, providing further evidence that subclinical congestion is associated with the extent of myocardial damage. We observed significant positive correlations between EVLW and cardiac biomarkers. Hs-TnT and CK-MB are established markers associated with infarct size and prognosis following primary PCI (20, 21), while NT-proBNP is associated with short-term prognosis and HF diagnosis (22, 23). Furthermore, we found that patients who experienced MACE had significantly increased EVLW compared to those without MACE, and EVLW was an independent predictor of in-hospital MACE. The imaging findings observed in patients with elevated EVLW—including subpleural ground-glass opacity, subpleural consolidation, pulmonary ground-glass opacity, and interlobular septal thickening—provide visual correlates of the increased lung water detected by quantitative CT analysis.

From a clinical perspective, the GRACE score is widely used for risk stratification in acute coronary syndrome and effectively predicts short-term mortality (5, 24–26). In our cohort, while the GRACE score was a significant predictor of MACE in the overall STEMI population, its predictive power was limited when applied exclusively to Killip class 1 patients. This limitation may be partly attributable to the clinical stability of these patients at admission (absence of cardiogenic shock or life-threatening arrhythmias) and the fact that clinically apparent pulmonary congestion (Killip class >1) is a major driver of GRACE's discriminatory ability (27, 28). We found that patients who experienced MACE had significantly increased EVLW compared to those without MACE, and EVLW was an independent predictor of in-hospital MACE. Importantly, the addition of EVLW to the GRACE score significantly enhanced its predictive ability for in-hospital MACE. This finding indicates that in STEMI patients classified as Killip class 1, the presence of subclinical congestion identifies a subset of patients at higher risk for adverse outcomes, and EVLW contributes unique prognostic information independent of the GRACE score. Clinically, this may help identify high-risk patients within this apparently low-risk group, enabling personalized treatment adjustments to optimize outcomes.

Another interesting finding of our study was the association between inflammatory markers and subclinical pulmonary congestion, which aligns with several previous studies (29, 30). Furthermore, hematocrit levels correlated with EVLW. A possible explanation is that alterations in hematocrit may influence alveolar-capillary interface conductance (13). The mechanisms underlying subclinical pulmonary congestion in STEMI patients with Killip class 1 remain unclear and warrant further investigation.

## Limitations

Nevertheless, the limitations of the current study should be acknowledged. Firstly, this investigation was a single-center retrospective study with a limited patient cohort because CT was not performed for every AMI patient, which may introduce selection bias. Secondly, the upper limit for the quantitative measurement of hs-TnT in our hospital is established at 10 ng/mL. Consequently, for samples where hs-TnT levels exceeded this threshold, the value was recorded as 10 ng/mL for analysis. Thirdly, NT-proBNP levels were not routinely obtained at admission for all patients, resulting in missing data for nine individuals. Fourth, although images with significant artifacts were excluded, subtle motion artifacts cannot be completely ruled out and may have minor effects on EVLW calculations. Additionally, potential confounding by inflammatory or pulmonary factors not fully captured by our exclusion criteria cannot be completely excluded. Finally, the mechanisms for subclinical pulmonary congestion in STEMI patients remain unclear and further studies are required to clarify the mechanisms.

## Conclusion

In conclusion, subclinical pulmonary congestion identified by CT in STEMI patients with Killip class 1 was associated with biomarkers of myocardial damage and in-hospital MACE. The EVLW enhanced the predictive ability of GRACE concerning the in-hospital MACE of STEMI patients. Despite the absence of clinically overt HF signs upon admission, the presence of subclinical congestion in STEMI patients is still associated with an even worse outcome, suggesting that these patients may require more aggressive intervention and careful monitoring.

## Data Availability

The raw data supporting the conclusions of this article will be made available by the authors, without undue reservation.
